# SYSTEMS BIOLOGY OF AGING

**DOI:** 10.1093/geroni/igac059.072

**Published:** 2022-12-20

**Authors:** Timothy Rhoads

## Abstract

Program Overview: Aging is a complex process; numerous aspects of cell and animal physiology change with age and pinpointing a single causal mechanism has proven difficult. A systems-level approach, which attempts to integrate multiple perspectives of aging into a more cohesive understanding, addresses this challenge by leveraging the explosion in recent years of molecular and biological “big data.” In this session, speakers will describe their efforts using systems biology approaches to achieve a multivariate assessment of the mechanisms of aging. Dr. Gladyshev will speak on recent work to understand the systemic mechanisms that underpin lifespan control. Dr. Levine moves the focus to epigenetic measures of aging, an emerging biomarker. Dr. Kane will describe efforts to examine frailty phenotypes and their biological underpinnings, as well as sexual dimorphism during aging in mice. Dr. Rhoads will describe the broad landscape of RNA processing, a mechanism with a recently described association with aging and delayed aging by caloric restriction, in multiple organisms and tissue contexts, as well as engagement of this mechanism during different aging interventions. Together our speakers advance our understanding of the multifactorial nature of aging biology and set the stage for the development of interventions that target all aspects of the aging process.

